# Factors Associated With Use of an Online Telemedicine Service to Access Self-managed Medical Abortion in the US

**DOI:** 10.1001/jamanetworkopen.2021.11852

**Published:** 2021-05-21

**Authors:** Abigail R. A. Aiken, Jennifer E. Starling, Rebecca Gomperts

**Affiliations:** 1 LBJ School of Public Affairs, The University of Texas at Austin; 2 Population Research Center, The University of Texas at Austin; 3 Mathematica Policy Research Inc, Cambridge, Massachusetts; 4 Aid Access, Amsterdam, the Netherlands

## Abstract

**Importance:**

People in the US have been seeking self-managed abortions outside the formal health care system using medications obtained through online telemedicine. However, little is known about this practice, including potential motivating factors.

**Objective:**

To examine individual reasons for accessing medication abortion through an online telemedicine service as well as associations between state- and county-level factors and the rate of requests.

**Design, Setting, and Participants:**

This population-based cross-sectional study examined all requests for self-managed medication abortion through an online consultation form available from Aid Access, a telemedicine service in the US, between March 20, 2018, and March 20, 2020.

**Main Outcomes and Measures:**

Individual-level reasons for accessing the telemedicine service were examined as well as the rate of requests per 100 000 women of reproductive age by state. Zip code data provided by individuals making requests were used to examine county-level factors hypothesized to be associated with increased demand for self-managed abortion: distance to a clinic (calculated using location data for US abortion clinics) and the population proportion identifying as a member of a racial/ethnic minority group, living below the federal poverty level, and having broadband internet access (calculated using census data).

**Results:**

During the 2-year study period, 57 506 individuals in 2458 counties in 50 states requested self-managed medication abortion; 52.1% were aged 20 to 29 years (mean [SD] age, 25.9 [6.7] years), 50.0% had children, and 99.9% were 10 weeks’ pregnant or less. The most common reasons cited by individuals making requests were the inability to afford in-clinic care (73.5%), privacy (49.3%), and clinic distance (40.4%). States with the highest rate of requests were Louisiana (202.7 per 100 000 women) and Mississippi (199.9 per 100 000 women). At the county level, an increase of 1 SD (47 miles) in distance to the nearest clinic was significantly associated with a 41% increase in requests (incidence rate ratio, 1.41; 95% CI, 1.31-1.51; *P* < .001), and a 10% increase in the population living below the federal poverty level was significantly associated with a 20% increase in requests (incidence rate ratio, 1.20; 95% CI, 1.13-1.28; *P* < .001).

**Conclusions and Relevance:**

In this cross-sectional study, clinic access barriers were the most commonly cited reason for requesting self-managed medication abortion using an online telemedicine service. At the county level, distance to an abortion clinic and living below the federal poverty level were associated with a higher rate of requests. State and federal legislation could address these access barriers.

## Key Points

**Question:**

What factors are associated with use of an online telemedicine service for accessing self-managed medication abortion in the US?

**Findings:**

In this cross-sectional study of 57 506 individuals in 2458 counties, the cost of in-clinic care was the most commonly cited reason for accessing self-managed abortion using online telemedicine. At the county level, a 47-mile increase in distance to the nearest clinic was significantly associated with a 41% increase in requests, and a 10% increase in the population living below the federal poverty level was significantly associated with a 20% increase in requests.

**Meaning:**

In this study, clinic access barriers were the most common reason for accessing self-managed medication abortion, and both distance to an abortion clinic and living below the federal poverty level were associated with higher demand for self-management.

## Teaser

This cross-sectional study assesses the reasons that individuals in the US are accessing self-managed medication abortion through online telemedicine services and the factors associated with these requests.

## Introduction

Self-managed medication abortion is the practice of using mifepristone and misoprostol or misoprostol alone to conduct a medication abortion outside the formal health care setting.^1^ This practice may be increasing in the US for 2 main reasons. First, in many states, access to abortion in the clinical setting is limited.^2^ At the state level, Targeted Regulation of Abortion Provider (TRAP) laws have forced clinics to close,^3^ and state-mandated requirements, such as pre-abortion ultrasonography, waiting periods, and parental consent have placed substantial and sometimes insurmountable burdens on individuals seeking clinical abortion care.^4,5^ At the federal level, the Hyde Amendment, which bans federal funding for almost all abortions, may put clinical care out of financial reach in states that do not use state Medicaid dollars to cover abortion care.^6^

Second, there is increasing evidence that medication abortion provided without ultrasonography and with minimal oversight from health care professionals is safe and effective.^7,8^ Since its approval, mifepristone has been subject to a risk evaluation management strategy (REMS), which restricts its use in ways not supported by evidence, including an in-person dispensing requirement. Health care professionals have called for the US Food and Drug Administration to remove the REMS and enable mifepristone to be obtained from pharmacies after remote consultation with a provider.^9^ Thus, an increasing number of individuals view self-managed medication abortion as part of the spectrum of abortion care.

Little is known, however, about who uses self-managed medication abortion in the US or the factors associated with the need or desire to self-manage. Insights have come from mixed-methods studies in Texas and from abortion clinics in major cities where individuals discuss self-management using a variety of methods and cite myriad reasons, including barriers to clinic access and personal preference.^10,11^ Few studies have engaged in quantitative analysis of self-management at the national level, including 1 study that assessed lifetime prevalence^12^ and another that assessed the demand for medication self-management and found that there was demand in every state.^13^

Until recently, access to self-managed medication abortion in the US has been mainly through pharmacies in Mexico, social networks, or online pharmacies.^14^ Online pharmacies are often viewed with suspicion by individuals seeking medication^5^ despite the medications usually being authentic.^15^ However, access changed markedly in 2018, when Aid Access, the first online telemedicine service to offer abortion medications to individuals living anywhere in the US, was launched.^16^ Using data from this service, we examined the individual-level characteristics and motivations of individuals who made requests for abortion medications, the rate of requests by state, and the factors associated with the rate of requests at the county level.

## Methods

This cross-sectional study examined all requests for a self-managed medication abortion through Aid Access between March 20, 2018, and March 20, 2020. Individuals make requests to Aid Access by filling out an online consultation form. A doctor reviews the form to check for contraindications and a reported gestation of 10 weeks or less. Mifepristone and misoprostol are then prescribed according to the World Health Organization–recommended medication abortion protocol^17^ and mailed by a partner organization. The Aid Access help desk is available to users at any time during and after an abortion. Trained help desk members provide email instructions for use of the medications and information about recognizing complications that may require in-person medical attention. Aid Access provided all data for this study in a fully deidentified format. Researchers had access only to the state and zip code aspects of mailing information. At the time of accessing the service, respondents consented to the fully anonymized use of their data for research purposes. Written informed consent was obtained from all individuals. The study received ethical approval from the institutional review board at The University of Texas at Austin. This study followed the Strengthening the Reporting of Observational Studies in Epidemiology (STROBE) reporting guideline.

The study dates represent the first 2 years of service operation, from official launch to the beginning of the COVID-19 pandemic in the US, which necessitated temporary changes to the service model. Consultation forms contained information about age, parity, state of residence, circumstances of pregnancy, gestation at the time of request, whether gestation had been determined by ultrasonography, reasons for not undergoing ultrasonography, contraindications to medication abortion (eg, allergy to medications, intrauterine device in place, chronic adrenal failure, and inherited porphyria), distance to a hospital, presence of a support person, and motivations for accessing the service. Those making the request could decline to answer any question that did not determine medical eligibility. These individuals also provided information about their location for mailing purposes.

Motivations for accessing the service were sought using the request, “Please share with us the reasons why you are requesting treatment through this online service.” Answer options were based on prior insights from qualitative and quantitative studies^5,11,18^ and included an “other” option for specifying motivations not explicitly listed. The options listed included cost of clinical services, distance to a clinic, difficulty finding childcare, difficulty taking time away from work or school, legal restrictions (such as being required to view ultrasonography findings), experienced or perceived stigma or judgment, intimidation or harassment by protestors, inability to maintain confidentiality (eg, preference to avoid informing family members) when accessing clinic services, fear of negative consequences from a controlling or abusive partner, the comfort of the home environment, preferring autonomy during the abortion process, feeling empowered by self-management, the privacy of the home environment, and the ability to have a support person present during the abortion. Respondents could choose as many motivations as they believed applied to their situation.

Reasons for not undergoing ultrasonography before making a request were sought using the request, “Please share with us why you did not obtain an ultrasound.” A range of answer options were provided, including inability to afford ultrasonography, certainty about gestation based on the date of the last menstrual period, fear of pregnancy being discovered by others by attending a clinic, inability to get to a clinic because of distance or lack of transportation, inability to take time off school or work to visit a clinic, and uncertainty about where to access ultrasonography. An “other” option was included to capture motivations not explicitly listed. Respondents could choose as many reasons as they believed applied to their situation.

In addition to examining characteristics and motivations at the individual level, we examined per capita requests to Aid Access at the state level. We measured the rate of requests in each state per 100 000 women of reproductive age to visualize heterogeneity in demand across states. We then conducted an analysis of factors associated with increased requests to Aid Access at the county level. We used zip code data from consultation forms to create a count of Aid Access requests per 10 000 women of reproductive age in each county, mapping zip codes to counties based on centroid latitude and longitude. The population of women of reproductive age in each county was obtained from US Census Bureau 2019 data,^19^ and reproductive age was defined as 15 to 44 years.

We also obtained information on county-level spatial and demographic covariates that we hypothesized might be associated with requests for medication self-management, including mean distance across zip codes in each county to the nearest abortion clinic and the proportion of the county’s population that had broadband internet access, was non-White, and lived below the federal poverty level (FPL). To determine the clinic distance for each county, we calculated the mean distance in miles from each zip code in the county to the zip code of the nearest abortion clinic using the Great Circle Distance Formula.^20^ We obtained information on abortion clinic locations using 3 separate sources that are updated regularly: ineedana.com (compiled by A Team Tech),^21^ the Abortion Care Network clinic list, and the University of California, San Francisco, abortion clinic database.^22^ We cross-referenced these data to ensure maximum inclusion and accuracy. We calculated the proportion of each county’s population with broadband internet access using open-source data from the Federal Communications Commission Mapping Broadband Health in America project ^23^ and the proportion of each county’s population of women of reproductive age identifying as members of a racial/ethnic minority group using the most recent available data (2019) from the US Census Bureau, Population Division.^19^ We calculated the proportion of the population living below the FPL in each county using data from the US Census Bureau’s Small Area Income and Poverty Estimates.^24^

### Statistical Analysis

To explore the factors associated with increased requests to Aid Access per 10 000 women of reproductive age compared with state-level mean requests, we fit a multilevel negative binomial model to the county-level request data (N = 2458), in which the response variable was the reproductive-age population–adjusted total request count for each county and the explanatory variables were the county-level spatial and demographic covariates described above. We selected variables using a stepwise procedure and used variance inflation factors to guard against collinearity. We also tested for interactions between each of these variables and used likelihood ratio tests to select the best model fit. To account for the distinct state contexts in which the counties are nested, including both state abortion policy context as defined by the Guttmacher Institute state policy classification system^25^ and other unmeasured state-specific factors, we allowed model intercepts to vary at the state level. For ease of interpretability, we rescaled the county-level regression coefficient estimates and 95% CIs to represent the expected change in requests associated with 1 SD in clinic distance (47 miles) and 10% change in the percentage of the population identifying as members of a racial/ethnic minority group, having broadband access, and living below the FPL.

We used R statistical software, version 3.5.3 (R Project for Statistical Computing) to conduct all data analysis and the mpath package to fit our models.^26^ We considered α < .05 to indicate statistical significance.

## Results

Between March 20, 2018, and March 20, 2020, 57 506 individuals requested a medication abortion from Aid Access; Table 1 shows their demographic and clinical characteristics. A total of 52.1% of these individuals were aged 20 to 29 years, and 19.6% were younger than 20 (mean [SD] age, 25.9 [6.7]); 50.0% had children. At the time requests were made, 99.9% reported being pregnant for 10 weeks or less; gestation was 6 weeks or less for 43.6%, between 7 and 10 weeks for 56.3%, and more than 10 weeks for 0.1%. A total of 53.5% had experienced a contraceptive failure. Most individuals (84.5%) had not undergone ultrasonography, with the most common reasons being inability to afford it (56.4%) and certainty about gestation based on date of last menstrual period (33.4%). Most individuals (96.3%) were within an hour’s drive to a hospital, and 96.4% had a companion who could be with them during the abortion. A total of 0.6% of individuals had any contraindication, and 99.1% felt sure about their decision.

**Table 1.** Characteristics of 57 506 Individuals Requesting Medication Abortion From Aid Access Between March 20, 2018, and March 20, 2020

Characteristic	Frequency of requests, No. (%)
Age, y	
<20	11 283 (19.6)
20-24	16 056 (27.9)
25-29	13 884 (24.1)
30-34	9317 (16.2)
35-39	5077 (8.8)
40-44	1606 (2.8)
≥45	283 (0.5)
Children, No.	
0	28 754 (50.0)
1	11 288 (19.6)
2	9592 (16.7)
≥3	7872 (13.7)
Gestation	
≤6 wk	25 100 (43.6)
7-10 wk	32 399 (56.3)
>10 wk	7 (0.1)
Circumstances of pregnancy^a^	
Contraceptive failure	30 766 (53.5)
Contraceptive nonuse	23 891 (41.6)
Sexual assault	2848 (4.9)
Ultrasonography performed before abortion request	
Yes	8895 (15.5)
No	48 611 (84.5)
Reason for no ultrasonography^b^	
Cannot afford to pay for one	27 436 (56.4)
Do not need one owing to feeling sure of gestation	16 244 (33.4)
Afraid someone will find out if trip to a clinic is made to get one	15 350 (31.6)
Cannot get to a clinic to get one^c^	11 557 (23.8)
No time to get one	4863 (10.0)
Not sure where to get one	4725 (9.7)
Other reason	1105 (2.3)
Contraindication to medication abortion	
Yes	340 (0.6)
No	57 166 (99.4)
Within a 60-min drive to a hospital	
Yes	55 383 (96.3)
No	2123 (3.7)
Another individual can be available and present during the abortion	
Yes	55 449 (96.4)
No	2057 (3.6)
Feelings about decision	
OK with decision	56 960 (99.1)
Troubled by decision	546 (0.9)

^a^ One missing response.

^b^ Respondents could choose more than 1 response.

^c^ Reasons include distance and lack of transportation.

**Figure 1.**
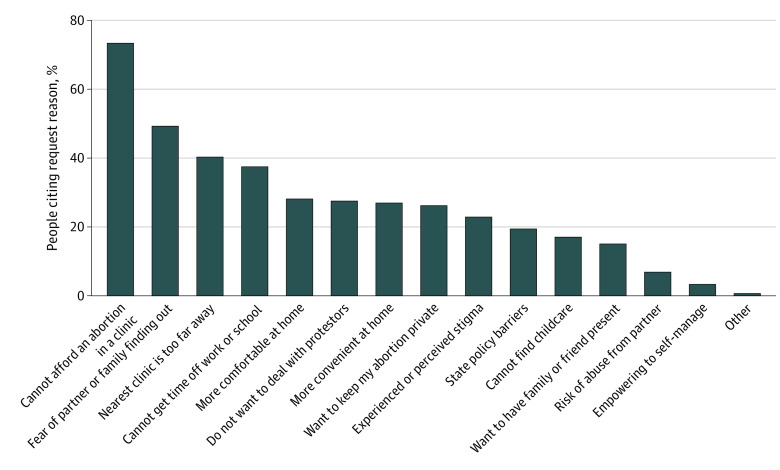
Reasons for Requesting Self-managed Medication Abortion Using an Online Telemedicine Service

The most common reason for seeking a medication abortion through Aid Access was inability to afford an in-clinic abortion, cited by 73.5% of individuals making requests (Figure 1). Almost half (49.3%) were afraid of a partner or family member finding out if they tried to go to a clinic, 40.4% reported that the nearest clinic was too far away, and 37.6% were unable to take time away from work or school to go to a clinic. Although the most common reasons involved barriers to clinic access, preferences for self-managed abortion were also cited: 28.2% responded that they would be more comfortable at home, and 27.0% said self-managing at home would be more convenient.

**Figure 2.**
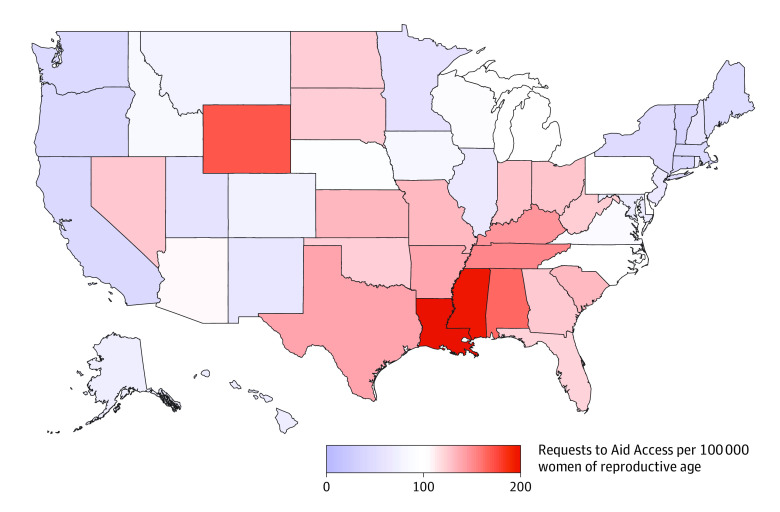
Rate of Requests to Aid Access for Abortion Medications by State Between March 20, 2018, and March 20, 2020

Requests to Aid Access came from all 50 states. Figure 2 shows the rate of requests by state per 100 000 women of reproductive age. Louisiana had the highest rate of requests (202.7 per 100 000 women), followed by Mississippi (199.9 per 100 000 women), Wyoming (173.2 per 100 000 women), and Alabama (166.1 per 100 000 women). Vermont had the lowest rate of requests (36.7 per 100 000 women), followed by Connecticut (41.8 per 100 000 women), Oregon (43.7 per 100 000 women), and California (44.6 per 100 000 women).

County-level factors associated with a higher rate of requests to Aid Access included distance to the nearest abortion clinic and the proportion of the population living below the FPL (Table 2). The mean (SD) distance to the nearest clinic for all counties was 56.5 (47.4) miles. A 47 mile (1 SD) increase in distance to the nearest clinic was associated with a 41.0% increase in Aid Access requests (incidence rate ratio [IRR], 1.41; 95% CI, 1.31-1.51; *P* < .001). A 10% increase in the population living below the FPL was associated with a 20.0% increase in requests (IRR, 1.20; 95% CI, 1.13-1.28; *P* < .001). The interaction between clinic distance and the proportion living below the FPL was associated with a 12.0% lower rate of requests (IRR, 0.88; 95% CI, 0.84-0.92; *P* < .001).

**Table 2.** County-Level Factors Associated With Requests to Aid Access

Covariate	IRR (95% CI)	*P* value
Clinic distance *z* score^a^	1.41 (1.31-1.51)	<.001
Proportion of population living below the FPL^b^	1.20 (1.13-1.28)	<.001
Proportion of population belonging to racial/ethnic minority group^b^	0.99 (0.95-1.02)	.50
Proportion of population with broadband internet access^b^	1.01 (1.00-1.02)	.06
Interaction between clinic distance and proportion of population living below the FPL^b^	0.88 (0.84-0.92)	<.001
Interaction between and proportion of population living below the FPL and belonging to racial/ethnic minority group^b^	0.92 (0.77-1.09)	.34

Abbreviations: FPL, federal poverty level; IRR, incidence rate ratio.

^a^ The mean (SD) distance to a clinic was 56 (47) miles. The estimate represents the expected associated change in requests for a 1-SD increase in clinic distance.

^b^ Adjusted so that the estimate represents the expected change in requests for a 0.1-change in proportion.

**Figure 3.**
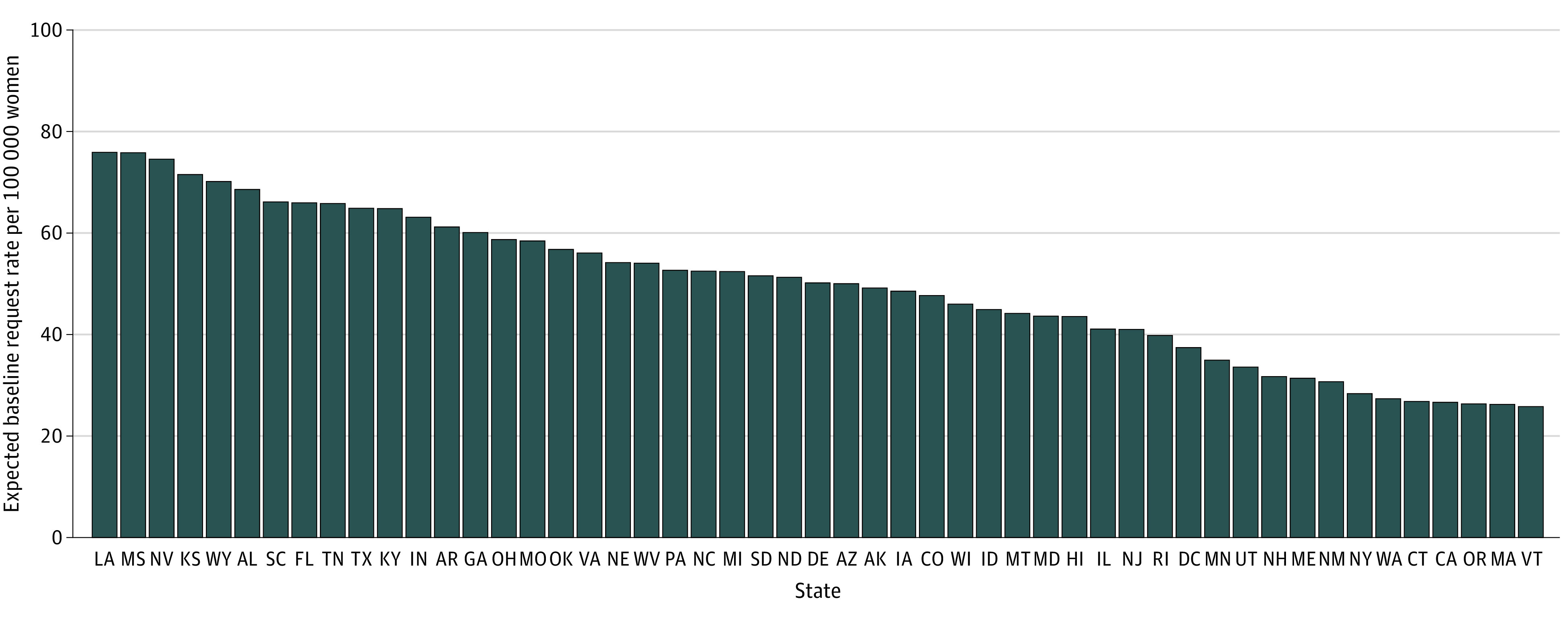
State-Level Intercepts in the Multilevel Negative Binomial Regression Model Baseline expected requests to Aid Access per 100 000 individuals of reproductive age under reference covariate levels are shown. Variability in intercepts (mean, 50.9; SD, 14.8) reflects state-specific policy and other differences that are not captured by covariates.

Figure 3 shows the magnitude of the state-level intercepts, which are scaled to represent the baseline expected request rate per 100 000 women of reproductive age for each state under the reference level for all other covariates in the model (ie, mean clinic distance and all county-level characteristics set to 0). The magnitude of the state-level intercepts (SD of the intercepts, 0.33) was larger in states with more restrictive abortion policy climates,^25^ with the top 5 being Louisiana (76.1), Mississippi (75.8), Nevada (74.7), Kansas (73.2), and Wyoming (71.0). The magnitude of the intercepts was smaller in states with more supportive policy climates,^25^ with the lowest being Vermont (27.0), Massachusetts (27.2), Oregon (27.6), California (28.5), and Connecticut (28.7).

## Discussion

To our knowledge, this study is the first to examine requests for self-managed medication abortion using an online telemedicine service in the US and the factors associated with these requests. We found that clinic access barriers, most notably the cost of an in-clinic abortion, were the reasons most frequently cited by those making requests. We also found that these individual-level barriers were reflected at the state level, where the highest rates of requests were associated with residence in states with more restrictive abortion policy climates, and at the county level, where longer mean distance to the nearest abortion clinic and a higher proportion living below the FPL were associated with an increased rate of requests.

In light of the declining abortion rate in the US, these findings lend support to the possibility that there has been a shift in the location of some abortions to outside the clinic setting and thus an increase in the number of self-managed abortions.^27^ Policies restricting access to in-clinic abortion have increased over the past decade,^28^ and some findings suggest that the decrease in the rate of in-clinic abortions has been associated with residence in states with more restrictions.^29^ Moreover, the number abortion clinics in the Midwest and South has decreased by 6% and 9%, respectively,^30^ resulting in increased distance to an abortion clinic and an associated decrease in the in-clinic abortion rate.^31^ These patterns mirror our findings that cost and distance to a clinic were associated with increased demand for self-management. The interaction examined between poverty and clinic distance also showed that although closer clinic distance was associated with a decrease in requests to Aid Access, this association did not hold for people living in below the FPL. In other words, in places where individuals could not afford the cost of an in-clinic abortion, a shorter distance to a clinic was not associated with improved access.

In addition to the difficulties exacerbated by living below the FPL, such as finding information, taking time away from work and childcare, and keeping multiple state-mandated clinic appointments, 1 of the most significant financial obstacles may be the lack of Medicaid coverage for abortion under the Hyde Amendment.^32^ Although state Medicaid funds can be used to pay for abortion care in 16 states, they cannot be used in 34 states,^33^ and Patient Protection and Affordable Care Act health insurance plans are prohibited from covering abortion in many of these same 34 states.^34^ Although the state-level intercepts in our model accounted for more than policy differences, we note that the states with restrictions on insurance and Medicaid coverage tended to have higher baseline request rates. These policies mean that most individuals must pay out of pocket for abortion care, and although abortion funds assist with these payments, our findings suggest that self-management also fills a gap in access.

Of note, barriers to clinic access were not the sole or primary motivation for all individuals. Some also cited preferences for the comfort and convenience of using abortion medications at home. Such preferences are also commonly reported in other countries where self-managed abortion is provided using online telemedicine services.^14^ Moreover, studies have demonstrated high levels of effectiveness and safety, with few reported serious adverse events.^7,14^ Self-managed abortion as a preference thus calls attention to the need for a wider spectrum of abortion care models that center on individual autonomy and preferences.

With many states planning further restrictions on in-clinic abortion and the possibility that *Roe v Wade* will be severely diminished by Supreme Court rulings, we may expect to see demand for self-managed medication abortion increase. The temporary adjustment to the mifepristone REMS during the COVID-19 pandemic has spurred some states to increase access to medication abortion by introducing no-test protocols, telemedicine consultation, and delivery of medications by mail.^35^ These innovations also have the potential to expand access for those living below the FPL by negating the costs of in-person clinic attendance and medical tests. However, other states have kept in place laws that prohibit telemedicine consultation and, in some cases, have moved to further restrict clinic access,^36^ with associated increases in demand for self-management.^37^ For clinicians, this increased demand for self-managed abortion means being equipped to provide follow-up to those patients who might require it, including being informed on the American College of Obstetricians and Gynecologists' position on self-management of abortion^38^ and the lack of reporting requirements to protect patient confidentiality.^39^ For policy makers, increased demand means that the consequences of laws that make in-clinic abortion less accessible and the expansion of medication abortion access should be considered to help overcome existing barriers and meet patients’ preferences.

### Limitations

This study has limitations. The main limitation of our study was that other routes to self-managed medication abortion and other types of self-management are available in the US. Therefore, our results may not be generalizable beyond the population using an online telemedicine service. Furthermore, our methodological approach lends itself to exploring associations rather than establishing causality. Nonetheless, we hope that future studies will examine in more detail the factors associated with higher rates of self-managed medication abortion that were revealed in the present study.

## Conclusions

In this cross-sectional study, clinic access barriers owing to cost and distance were the most commonly cited barriers by individuals accessing self-managed medication abortion using an online telemedicine service. At the county level, longer distance to an abortion clinic and living below the FPL were associated with increased demand for the service. At the state level, the highest rates of requests to the service were found in states with more restrictive abortion policy climates. Repeal of the Hyde Amendment and permanent removal of the mifepristone REMS may help to address these barriers.
